# Claudin 1 expression in basal-like breast cancer is related to patient age

**DOI:** 10.1186/1471-2407-13-268

**Published:** 2013-05-30

**Authors:** Anne A Blanchard, Xiuli Ma, Kevin J Dueck, Carla Penner, Steven C Cooper, Drew Mulhall, Leigh C Murphy, Etienne Leygue, Yvonne Myal

**Affiliations:** 1 Department of Pathology, University of Manitoba, 770 Bannatyne Avenue, Winnipeg, Manitoba R3E0W3, Canada; 2 Department of Physiology, University of Manitoba, Winnipeg, Manitoba, Canada; 3 Department of Biochemistry and Medical Genetics, University of Manitoba, Winnipeg, Manitoba, Canada; 4 Manitoba Institute of Cell Biology, Winnipeg, Manitoba, Canada

**Keywords:** Claudin 1, Tight junction protein, Basal-like breast cancer, Age, Tissue microarray

## Abstract

**Background:**

Defects in tight junctions, gate-keepers of the integrity of the epidermal barrier function, are known to contribute to cancer development. As such, enhancing our understanding of how the expression of proteins involved in these junctions is regulated in cancer, remains a priority. Although the expression of one of these proteins, claudin 1, is down regulated in most invasive human breast cancers (HBC), we have recently shown that high levels of claudin 1, characterized tumors belonging to the very aggressive basal-like breast cancer (BLBC) subtype. In these tumors, the claudin 1 protein, usually localized in the cell membrane, is often mislocalized to the cytoplasm.

**Methods:**

To examine the clinical relevance of this observation, we have generated and analyzed an invasive HBC tissue microarray consisting of 151 breast tumor samples; 79 of which presented a basal-like phenotype (i.e. ER-ve, PR-ve HER2-ve, CK5/6 or EGFR+ve). We also interrogated the outcome of claudin 1 knockdown in a human BLBC cell line, BT-20.

**Results:**

Immunohistochemical analysis of this patient cohort revealed a significant association between high claudin 1 expression and BLBCs in women 55 years of age and older. Interestingly, no significant association was found between claudin 1 and nodal involvement, tumor grade or tumor size. Regression analysis however, showed a significant positive association between claudin 1 and claudin 4, even though claudin 4 did not significantly correlate with patient age. Claudin 1 knockdown in BT-20 cells resulted in decreased cell migration. It also significantly altered the expression of several genes involved in epithelial-mesenchymal-transition (EMT); in particular, SERPINE 1 (PAI1) and SSP1 (osteopontin), known to inhibit EMT and cancer cell migration. Conversely, genes known to maintain EMT through their interaction, SNAIL2, TCF4 and FOXC2 were significantly down regulated.

**Conclusions:**

The association of high claudin 1 protein levels observed in tumors derived from older women with BLBC, suggests that claudin 1 has the potential to serve as a marker which can identify a specific subgroup of patients within the BLBC subtype and thus, further contribute to the characterization of these ill-defined breast cancers. More importantly, our studies strongly suggest that claudin 1 directly participates in promoting breast cancer progression, possibly through the alteration of expression of EMT genes.

## Background

A growing understanding of the heterogeneous nature of breast cancer has stemmed primarily from gene expression analysis studies, and more recently, integrated analysis of copy number and exome sequencing [1]. This has led to a redefinition of breast cancer subsets [1]. This new classification of breast cancer subtypes, focused on 10 genetically distinct groups, confirmed the prevalence of four previously identified molecular subtypes (luminal A, luminal B, HER2 +ve and the basal-like) [1]. Whereas the luminal A and B subtypes are characterized by their epithelial phenotypes, hormone sensitivity (estrogen receptor positive, ER+/ progesterone receptor positive, PR+), mildly invasive capacity and relatively good clinical outcome, the HER2+ and basal-like breast cancer (BLBC) subtypes are characterized by their mesenchymal phenotype, insensitivity to hormonal therapy (ER-ve; PR-ve), enhanced invasiveness and metastatic capacity [2] and poor clinical outcome [3-7].

The claudins belong to a family of tight junction (TJ) proteins (24 identified to date), that are crucial for the organization of epithelial cell polarity [8]. They contribute to the trans-epithelial barrier that controls the transport of ions and small molecules. They are also considered essential for the overall maintenance of the differentiated state of epithelial cells [9,10]. The claudins share a very distinct transmembrane topology: each family member is predicted to possess four transmembrane domains with intracellular amino and carboxyl-termini in the cytoplasm and two extracellular loops [11,12]. The expression pattern of the claudins is usually tissue specific; however, most tissues express multiple claudins that can interact in either a homotypic or heterotypic fashion to form the TJ strand. As well, the exact combination of claudin proteins within a given tissue determines the selectivity, strength and tightness of the TJ [11]. The claudins are also capable of recruiting signaling proteins, thereby regulating various cellular processes including cell growth, differentiation and tumorigenesis [13,14].

Claudin 1, the first member of this family to be identified, forms the backbone of the TJ strands and is crucial for the epidermal barrier function [15]. In cancer, an absence of, or defects in tight junctions have been associated with the development of the neoplastic phenotype. Although long suspected to play an active role in tumorigenesis, only recently have a number of studies demonstrated that claudin 1 directly participates in the progression of several cancers including melanomas [16], oral squamous cell carcinomas [17] and colon cancers [18].

Studies from our laboratory [19] and others [20-22] point toward a putative tumor suppressor role of claudin 1 in breast cancer as it is frequently down regulated in human invasive breast cancer and its absence or the down regulation of its expression is associated with poor prognosis [23]. We have however, also found high claudin 1 and claudin 4 protein expression associated with the BLBC subtype [19]. The BLBCs correspond to a subgroup of breast cancers that are poorly characterized and thus, mostly insensitive to most classical therapeutic strategies. Although a large cohort of human invasive breast cancers (350 samples) was examined in this earlier study, these tumors were of mixed pathological lesions (ductal, lobular, medullary, papillary, metaplastic), and of these, only 18 were of the BLBC subtype. As such, the clinical relevance of claudin 1 expression to the BLBCs could not be fully addressed.

The present study was carried out to determine whether the observed significant association between claudin 1 and the BLBC subtype could be clinically relevant. Specifically, we wanted to address whether there was an association between high levels of claudin 1 and disease recurrence and patient survival. However, since generally <15% of breast cancers are basal-like [24], the construction of a BLBC enriched tissue microarray (TMA) warranted the screening of a large number of tissue specimens. Thus, our strategy was to first pre-select tumors that were ER-ve and PR-ve (previously carried out by the ligand binding assay) and then identify those tumors that exhibited HER2 negativity as well as EGFR or CK5/6 positivity by immunohistochemistry (IHC). Seventy-nine out of 151 tumors were confirmed to be “basal-like” in our basal-like enriched TMA. Additionally, *in vitro* studies were carried out to examine whether claudin 1 had a direct functional role in human breast cancer. For these studies we used the human breast cancer cell line, BT-20 which is both phenotypically basal-like [25,26] and endogenously expresses high levels of this protein. Altogether this study provides evidence that claudin 1 identifies a specific subgroup of BLBC patients. We also demonstrate that claudin 1 could directly contribute to breast cancer progression.

## Methods

### Tissue microarrays

All invasive breast cancers used in the present study were obtained from the Manitoba Breast Tumour Bank (MBTB, University of Manitoba), which operates with the approval from the Faculty of Medicine, University of Manitoba, Research Ethics Board. As well the studies reported in this manuscript have been performed with the approval of the Bannatyne Campus, University of Manitoba, Research Ethics Board. Collection, handling and histo-pathological assessment of tumor tissues have been previously described [27,28]. The breast cancer tissue microarray (TMA) was constructed by the MBTB using a cohort of 151 breast tumor samples, which were determined to be estrogen receptor negative (ER-ve), progesterone receptor negative (PR-ve) by the ligand binding assay (ER-ve <3 fmol/mg protein, PR-ve <10 fmol/mg protein). Further, using a strict criteria for the basal-like subtype (ER-ve, PR-ve, HER2-ve and EGFR and/or CK5/6 +ve), 79 tumors were identified by IHC as having the BLBC phenotype. The remaining 72 tumors were designated as “non-basal”. The clinico-pathological characteristics of the patient cohorts were provided by the MBTB and used for statistical analyses.

### Immunohistochemical analysis of TMAs

IHC was performed as described previously on the BLBC enriched TMA [28]. Briefly, serial sections (5 μm) of the TMAs were stained with rabbit polyclonal antibodies to claudin 1 at a dilution of 1:150 (Life Technologies Inc., Burlington, ON, Canada), or claudin 4 at a dilution of 1:1200 (Abcam, Toronto, ON, Canada). The paraffin-embedded tissue sections were processed using an automated Discovery Staining Module, Ventana System (Tucson, AR, USA). Tissues were processed and incubated for 60 minutes with the primary antibody and 30 minutes with the secondary antibody following standard protocol. Validation of claudin 1 and claudin 4 antibodies has also been described previously [19]. Antibodies to CK5/6 (D5/16B4, Life Technologies Inc.), EGFR (3C6, Ventana Systems), and HER2 (Cb11, NovaCastra, Concord, ON, Canada) were used as previously detailed [28]. The TMA consisted of a total of 151 human invasive breast tumor biopsies, however only those tumors from which we were able to retrieve interpretable data (intact, unfolded tumor sections) were considered for our analysis. The IHC data, compiled into the database maintained by the MBTB, was made available for correlation analyses and other statistical comparisons [27,29].

### Quantification and cut-off selection

Positive staining was assessed by light microscopy. A semi-quantitative assessment was used. Both staining intensity (scale 0–3) and the percentage of positive cells (0-100%) were multiplied to generate an H score ranging from 0–300, as previously described [27,28]. TMA staining was evaluated independently by two investigators AB and CP. Where discordance (i.e. different scores given by different investigators) was found, cases were re-evaluated commonly and a consensus reached. Only tumor biopsies whose ER/PR status was determined by both ligand-binding assay (ER-ve <3 fmol/mg protein, PR-ve <10 fmol/mg protein), and by IHC (ER-ve/PR-ve <10% positive cells) were considered as negative in this study. Primary categorical analysis was carried out as follows: positivity for CK5/6 and EGFR was set as ≥10% of cells staining, and for HER2, tumor cores that showed membrane-staining intensity of 2 or 3 were considered positive.

### Human breast cancer cell lines and cell culture

The HBC cell line BT-20 was obtained from the American Type Culture Collection (ATCC, Manassas, VA, USA). Cells were cultured in Eagle’s Minimum Essential Medium (EMEM, Hyclone Laboratories Inc., Logan UT, USA) with 10% fetal bovine serum (PAA Laboratories Inc. Etobicoke, ON, Canada) supplemented with 100 units/mL penicillin, 100 mg/mL streptomycin, and 1mM pyruvate. Cells were grown at 37°C in an atmosphere of 95% air and 5% CO_2_.

### Generation of stable claudin 1 knockdown clonal cell lines

BT-20 cells were stably transfected with a SureSilencing shRNA control sequence plasmid (SA Biosciences Corporation, Frederick, MD, USA), and two different shRNA sequences (sequence 3 and 4; SA Biosciences) specific for the claudin 1 gene using Lipofectamine 2000 (Life Technologies Inc,). Single clones were selected using Hygromycin B (Life Technologies, Inc.), and knockdown of claudin 1 was confirmed by Western blot analysis.

### Subcellular fractionation

BT-20 cells were grown to 80% confluency and subcellular fractions were isolated using the ProteoExtract® Subcellular Proteome Extraction Kit (S-PEK, Calbiochem, Billerica, MA, USA) according to the manufacturer’s instructions. Protein fractions were subjected to acetone precipitation and pellets were reconstituted in sample isolation buffer (50 mM Tris-Cl pH 6.8: 5% SDS: 5mM β-glycerophosphate, containing complete mini protease inhibitor cocktail, Roche Diagnostics, Mississauga, ON, Canada). The mini BCA assay (ThermoScientific, Ottawa, ON, Canada) was used to determine the protein concentration of each fraction, prior to equal loading in 15% SDS-polyacrylamide electrophoresis gel and Western blotting.

### Wound healing/migration assay

BT-20 cells were grown to full confluency on 6-well plates and a scratch was made through the cell monolayer using a pipette tip. After washing twice with PBS, fresh tissue culture medium was added and photographs (ScopePhoto 3.0, ScopeTek DCM130 microscope camera) of wounded areas were taken in a time-dependent manner up to 18 hours after making the scratch. Measurements of the wound area were evaluated using the Image-J program (National Institutes of Health).

### Western blot analysis

Cells were lysed in an isolation buffer (50 mM Tris-Cl pH 6.8: 5% SDS: 5mM β-glycerophosphate, containing a complete mini protease inhibitor cocktail, Roche Diagnostics, Laval, QC, Canada) and mixed 3:1 with 4X sodium dodecyl sulfate (SDS) buffer [(500 mM Tris, pH 6.8), 40% glycerol, 8% SDS, 0.04% (w/v) bromophenol blue and 0.4M dithiothreitol (DTT)]. The samples were boiled for 5 min. at 100°C and electrophoresed in 15% SDS-polyacrylamide electrophoresis gel. Proteins were transferred to nitrocellulose, membranes were blocked in 5% non-fat milk in Tris-buffered saline with 0.05% Tween-20 (TBS-T) for 1 hr. Membranes were then incubated overnight at 4°C with primary antibodies (claudin 1, Life Technologies Inc.; β-actin, Abcam) diluted 1:1000, and 1:5000 respectively in blocking solution. Subsequently, the membranes were washed with TBS-T (three times 10 min.) and incubated with goat anti-rabbit or goat anti-mouse immunoglobulin G horseradish peroxidase conjugate (1:10000; Bio-Rad Laboratories Inc.) for 1 hr. at room temperature. The membrane was washed with TBS-T (three times 10 min.) and developed with Pico chemiluminescence substrate (Pierce Biotechnology, Rockford, IL, USA).

### Fluorescent microscopy

For immunofluorescence staining, BT-20 cells were cultured on glass cover slips and fixed with 100% methanol for 20 min at -20°C. Cover slips were then rinsed with PBS and the cells were permeabilized with 0.2% Tween-20 in PBS for 5 min., followed by three 20 min. washes with PBS. After blocking with 1% BSA in PBS for one hour at room temperature, cells were incubated with the claudin 1 rabbit primary antibody (Life Technologies Inc., dilution 1:50) overnight at 4°C in a humid chamber. The cells were washed three times for 10 min. with PBS and incubated with secondary anti-rabbit antibody conjugated with Cy3 (dilution 1:100) for one hour at room temperature. Cells were washed again with PBS, incubated with 4′, 6-diamidino-2-phenylindole-dihydrochloride (DAPI) and mounted in FluorSave (Calbiochem).

### Real-time PCR arrays

Cells were grown in EMEM in 6-well plates until 75-85% confluent and directly lysed by adding 350 uL Buffer RTL Plus from the RNeasy RNA extraction kit (Qiagen Sciences, Mississauga, ON, Canada). Equal amounts of RNA from two control clones were pooled and compared in triplicate with RNA from two claudin 1 knockdown clones. RNA (1μg/reaction) was reverse transcribed using the RT^2^ First Strand Kit (SA Biosciences Corporation). cDNA samples (25ng) were applied to each real-time PCR reaction on the human EMT RT^2^ Profiler PCR array (SA Biosciences Corporation) containing 84 key genes that change their expression during EMT. Real time PCR was carried out using the iCycler (BioRad Laboratories). The cycle profile consisted of denaturation at 95°C for 10 min., followed by 40 cycles of 95°C for 15 secs. and 60°C for 1 min. The iCycler iQ Optical System Software Version 3.0a (BioRad Resource Guide) was used to determine the cycle threshold (C_T_) for each reaction. Data was analyzed using the web-based PCR Array Data Analysis Software (SA Biosciences Corporation; http://www.sabiosciences.com/pcrarraydataanalysis.php). Five housekeeping genes were used as controls.

### Statistical analysis

Analysis was carried out as previously described [27,28], using SAS 9.2 (SAS, Cary, NC) statistical software. The Wilcoxon Two Sample test and the Kruskal-Wallis test were used to interrogate claudin l levels in tumor subtypes and tumors from different age groups of patients. Associations between claudin 1 and other clinical-pathological variables were tested using contingency methods (continuity adjusted Chi-Square was used for node, age and size data; Exact Linear Association was used for grade). Linear regression analyses with claudin 1 levels as dependent were also carried out. Univariate survival analyses were performed using Cox regression to generate Kaplan-Meier curves. Overall survival (OS) was defined as the time from initial surgery to the date of death attributable to breast cancer only. Recurrence time was defined as the time from initial surgery to the date of clinically documented local or distant disease recurrence. Analysis of Variance (ANOVA) followed by Bonferroni’s Multiple Comparison Test were used to assess differences in migration rates in the wound healing assays.

## Results

### High level of claudin 1 protein is associated with BLBCs derived from older women

**Figure 1:**
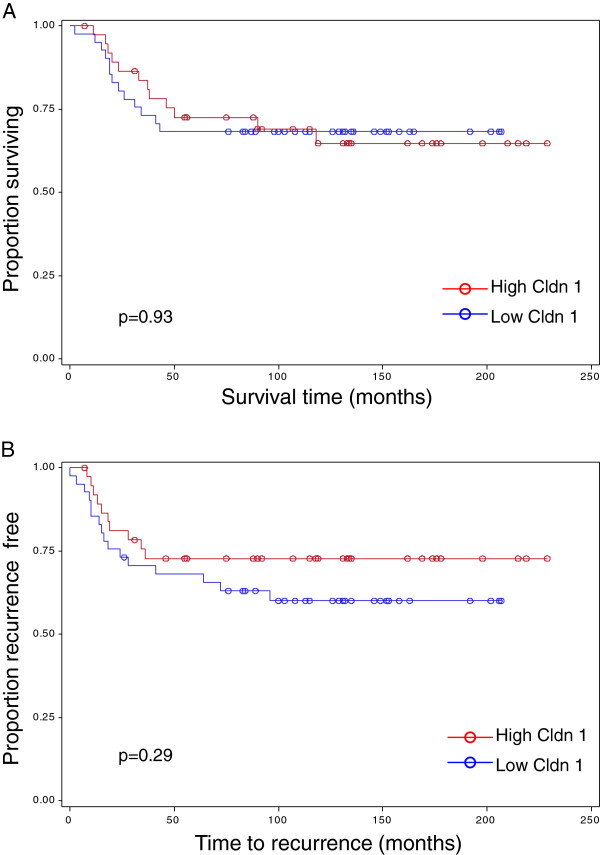
**Kaplan-Meier graphs for survival and recurrence in basal-like tumors.** Univariate survival analyses were performed using Cox regression. Symbols on the graph lines represent censored data. No significant association was found between claudin 1 expression and patient survival (p=0.93), nor recurrence of the disease (p=0.29); although a trend appeared towards significance for disease recurrence. **A**. Survival *n* = 79; low claudin 1 (H≤40) events = 13, high claudin 1(H>40) events = 12; **B**. Recurrence *n* = 79; low claudin 1 (H≤40) events = 16, high claudin 1(H>40) = 10. Cldn1 = claudin 1.

Claudin 1 expression was higher in the basal-like tumors compared to the non-basal tumors, confirming the observations made in our previous study [19]. A significantly higher median H-score (40) was associated with the basal-like tumors (n=79) versus the median H-score (20) of the non-basal tumors (n=72; p=0.02; Wilcoxon two sample test; Table 1). When both non-basal and basal-like tumors were included in the analysis, tumors originating from patients 55 years of age and older were more likely to have a higher median score for claudin 1 (H-score =55) than tumors derived from younger patients (p=0.06, Table 1). Overall, the highest level of claudin 1 protein expression was observed in the tumors from patients with BLBC who were older than 55 years of age (median H-score=90, p=0.004, Table 1). While a significant association between patient age and claudin 1 expression was observed in the BLBC group, no such association was observed with any other clinical parameter. Claudin 1 levels did not correlate with nodal status (p=0.21), tumor grade (p=0.92), nor tumor size (p=1.0, Table 2). Similarly, no significant association was found between claudin 1 expression and patient survival (p=0.93), nor recurrence of the disease (p=0.29); although a trend appeared towards significance for disease recurrence (Figure 1). EGFR and CK5/6, both markers for the BLBC phenotype, were found to be predictive for claudin 1 expression in the non-basal tumors (Table 3, p<0.0001, p=0.0007 respectively) but not in the basal-like tumors (p=0.12, p=0.20 respectively).

**Table 1:** Expression of claudin 1 and 4 in the combined basal-like and non-basal human invasive breast cancer cohort

		**Claudin 1**				**Claudin 4**			
		**n**	**Mean**	**Median**	***p *value**^**1**^	**n**	**Mean**	**Median**	***p *value**^**1**^
Age	≤55 yr	64	48.6	20	0.063	59	41.2	20	0.70
	> 55 yr	80	81.1	55		76	51.5	35	
Basal	no	65	54.8	20	0.017*	62	41.8	17.5	0.18
	yes	79	76.4	40		73	51.4	40	
Group	a) non-basal ≤55 yr	22	44.5	15	0.0036**	19	27.9	10	0.46
	b) non-basal > 55 yr	43	60.1	20		43	48.0	20	
	c) Basal ≤55 yr	42	50.7	25		40	47.5	35	
	d) Basal >55 yr	37	105.5	90		33	56.1	40	

^1^Statistical test Wilcoxon or Kruskal-Wallis for groups. * Statistically significant, p<0.05; ** p<0.01.

**Table 2:** Claudin 1 but not claudin 4 expression in basal-like breast tumors was significantly associated with patient age

**Clinical parameters**	**Subgroup cutoffs**	**Claudin 1**				**Claudin 4**			
		**High H-score >40**	**Low H-score ≤40**	**n**	***p *value**^**1**^	**High H-score >25**	**Low H-score ≤25**	**n**	***p *value**^**1**^
Node	+ve	18	13	31	0.21	15	14	29	0.64
	-ve	17	25	42		23	15	38	
Age	>55	23	14	37	0.034 *	20	13	33	0.81
	≤55	15	27	42		22	18	40	
Grade	low (3–5)	3	0	3	0.92	3	0	3	0.45
	moderate (6–7)	25	33	58		27	27	54	
	high (8–9)	10	8	18		12	4	16	
Size	>2cm	27	31	58	1	32	23	55	1
	≤2cm	8	10	18		9	7	16	

Breast tumors derived from women >55 years of age, had the highest claudin 1 levels. Expression was not significantly associated with other parameters examined.

^1^Continuity adjusted Chi-Square was used for node, age and size data, Exact Linear Association was used for grade. * Statistically significant, p<0.05.

**Table 3:** Linear regression analysis with claudin 1 protein levels as dependent

	**Basal-like tumors**			**Non-basal tumors**		
**Independent single predictor**	**Slope**	**n**	***p *value**	**Slope**	**n**	***p *value**
EGFR	0.503	79	0.12	1.040	60	<.0001
CK5/6	0.499	79	0.20	1.288	63	0.0007
Age	1.249	79	0.028	-0.520	65	0.40
Claudin 4	0.341	73	0.032	0.426	59	0.017

There was a significant association between claudin 1 and claudin 4 protein expression in both the basal-like (p=0.032) and non-basal (p=0.017) tumors (Table 3). However, claudin 4 protein level was not significantly associated with patient age (Table 1). Moreover, as with claudin 1, the protein expression of claudin 4 was also found not to be related to nodal status, size of the tumors nor tumor grade (Tables 1 and 2). However, there was a trend towards higher expression of claudin 4 in the BLBC, although not statistically significant (p=0.18, Table 1).

### Loss of membrane-associated claudin 1 protein in the BLBC

**Figure 2:**
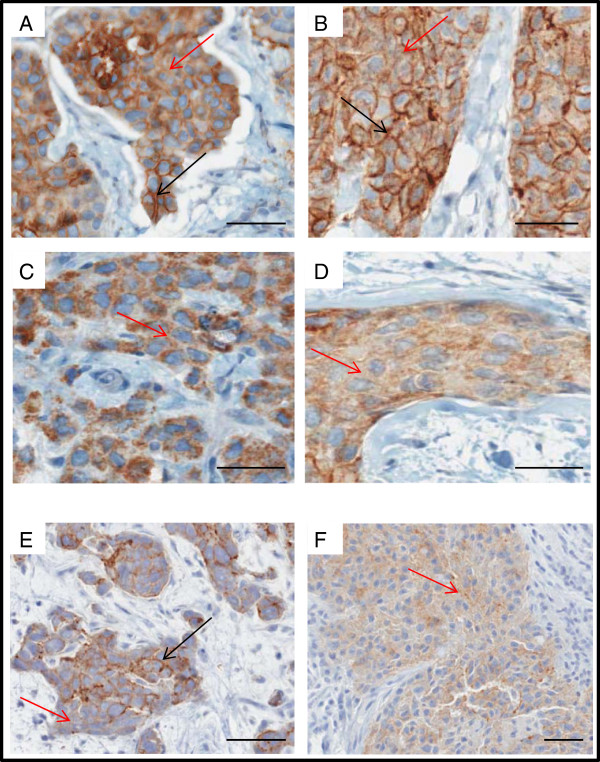
**Localization of claudin 1 and claudin 4 proteins in human invasive breast cancers. A**,**B**: Tumors showing both membrane and cytoplasmic staining with the claudin 1 antibody. **C**,**D**: Tumors showing cytoplasmic staining alone with the claudin 1 antibody **E**: Tumor showing both membrane and cytoplasmic staining with the claudin 4 antibody. **F**: Tumor showing cytoplasmic staining alone with the claudin 4 antibody (black arrows, membrane staining; red arrows, cytoplasmic staining). Scale bars represent 50μm.

Our results also showed membranous staining as well as cytoplasmic staining for claudin 1 in the breast tumors analyzed in the TMA (Figure 2). Some tumors cells exhibited membrane staining alone, cytoplasmic staining alone, or both cytoplasmic and membranous staining. Of the 79 basal-like tumors, 1 tumor was negative for both membranous and cytoplasmic staining, 11 tumors exhibited no membrane staining in any cells, while 67 tumors showed partial membrane staining, 51 of these in 10% or more tumor cells. The median percentage of tumor cells with membrane stain was 10%, whereas the median percentage of combined membrane and cytoplasmic staining was 30%, suggesting that a decrease in membrane staining resulted in an increase in cells in which claudin 1 was evident only in the cytoplasm. Patients whose tumors retained membrane claudin 1 expression in more than 10% of the tumor cells showed a trend towards increased survival (Kaplan-Meier analysis, p=0.25). As observed with claudin 1, claudin 4 was also more prevalent in the cytoplasm of the tumor cells (Figure 2).

### Claudin 1 is expressed in the membrane of BT-20 HBC cells

**Figure 3:**
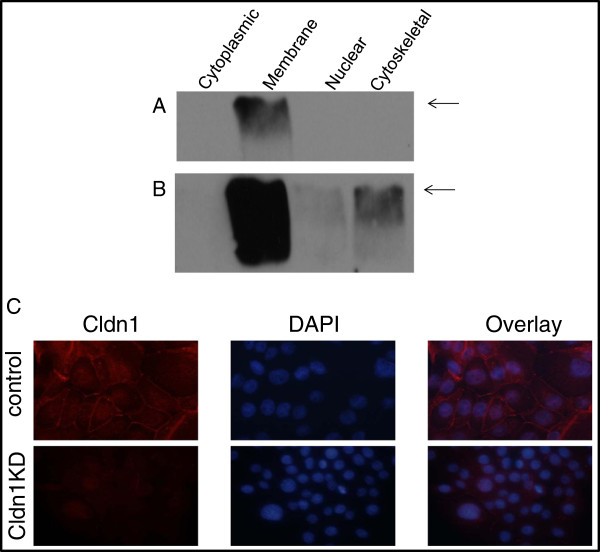
**Subcellular localization of claudin 1 protein in BT-20 cells.** Subcellular fractions of control BT-20 cells were analyzed by Western blot using the claudin 1 antibody. **A**. Short exposure shows claudin 1 in the membrane fraction only, **B**. longer exposure reveals some protein in the cytoskeletal and to a lesser extent, the nuclear fraction. The arrow indicates the 21kD claudin 1 protein. **C**. Immunofluorescent staining with the claudin 1 antibody (left panels) shows positive fluorescence for claudin 1 in the cell membrane and the cytoplasm of a control clonal cell line and reduced fluorescence in the claudin 1 knockdown clone (clone 3).

BT-20 is a BLBC cell line [25] which exhibits high endogenous levels of claudin 1. Subcellular fractionation studies were carried out to establish the localization of claudin 1 in these cells. Claudin 1 was primarily localized in the cell membrane component (Figure 3). Longer exposure revealed the presence of lower levels of claudin 1 in the cytoskeletal fraction and less so in the nuclear fraction (Figure 3A, B). This localization to the cell membrane was confirmed by IHC (Figure 3C).

### Identification and characterization of BT-20 claudin 1 knockdown clones

**Figure 4:**
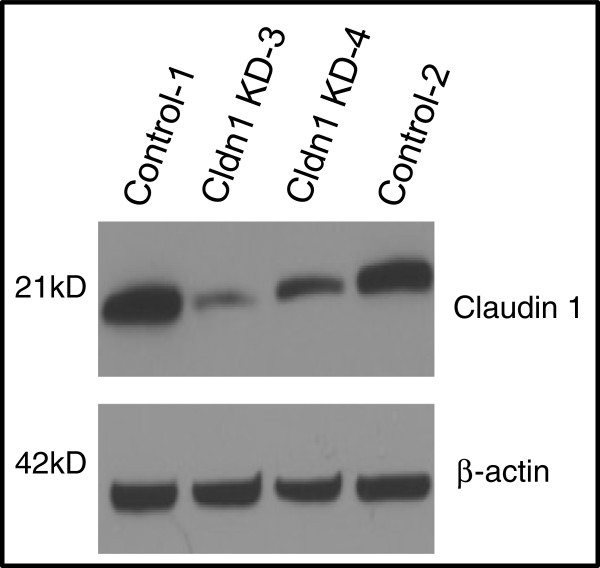
**Knockdown of claudin 1 protein in stably transfected BT-20 HBC cells.** Cells were transfected with a control sequence (C1, C2) and two different shRNA constructs targeting claudin 1 (3,4). The shRNA sequence 4 shows partial knockdown whereas sequence 3 shows >90% knockdown of the claudin 1 protein. (β-actin loading control; Western blot analysis; Cldn1 = claudin 1).

To delineate the loss of claudin 1 function in the BT-20 HBC cells, cells were stably transfected with claudin 1 shRNA constructs as described in the “Methods” section. Several clones exhibiting various levels of claudin 1 knockdown were characterized by Western blotting (Figure 4). Two clones, clones 3 and clone 4, transfected with two different claudin 1 targeting sequences, were selected for further studies. Clone 3 exhibited approximately 90% decrease in claudin 1 expression and about 70% knockdown was achieved for clone 4 compared to controls. Immunofluorescence (IF) analysis of the clonal lines show reduced level of claudin 1 in the cell membrane (Figure 3C) following claudin 1 knockdown.

### Knocking-down claudin 1 expression decreases cell migration

**Figure 5:**
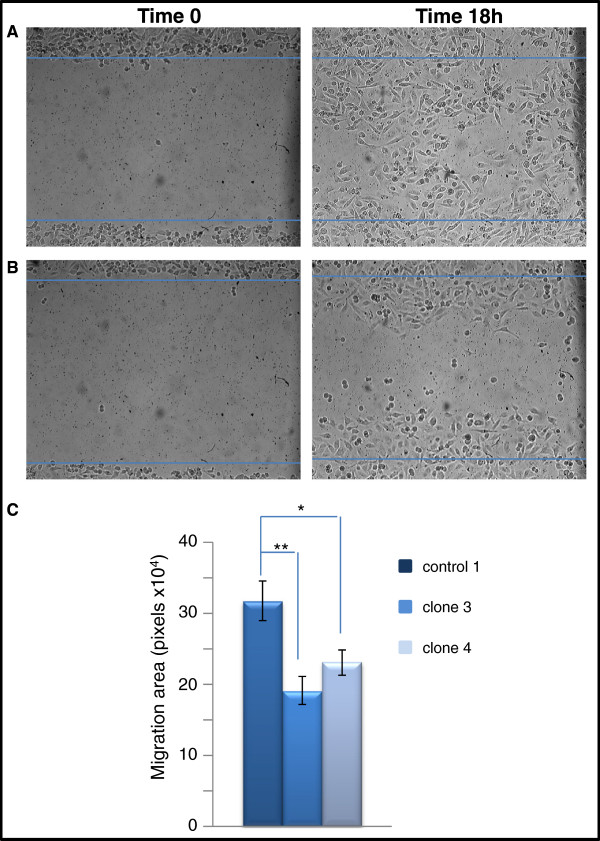
**Claudin 1 knockdown results in a decrease in cell migration rate in the BT-20 HBC cell line.** Representative light microscopic images of wound healing assays for claudin 1 knocked-down and control BT-20 cells used in evaluating migration rate into a cell free area are shown. Cells were grown to confluency and a scratch made through the cell monolayer. Measurements of the wound areas at time 0 (left panels) and 18h (right panels) were compared using the Image-J program which measured the surface area covered by migrating cells. **A**. BT-20 cells stably transfected with the control sh-RNA sequence; **B**. BT-20 cells stably transfected with the sh-RNA-claudin 1 vector. **C**. BT-20 cells stably transfected with a control shRNA sequence (control 1, n=12) migrated faster than the claudin 1 knockdown clones (clone 3, n=8; clone 4, n=12; mean ± S.E; ANOVA p=0.0054).* p<0.05, **p,0.01 Bonferroni’s Multiple Comparison Test.

To ascertain whether claudin 1 had a direct effect on cell migration and motility, claudin 1 knockdown cells were assayed using a monolayer wound-healing assay. In the knockdown clones, inhibition of claudin 1 resulted in a significant decrease (p<0.01, p<0.05, clone 3 and clone 4 respectively) in migration rate compared to controls (Figure 5). We observed that the clonal line 3, which exhibited a higher level (90%) of claudin 1 knockdown than clonal line 4 (70%) migrated at a slower rate than clone 4.

### Knocking-down claudin 1 expression alters the expression of genes associated with epithelial-mesenchymal transition

**Figure 6:**
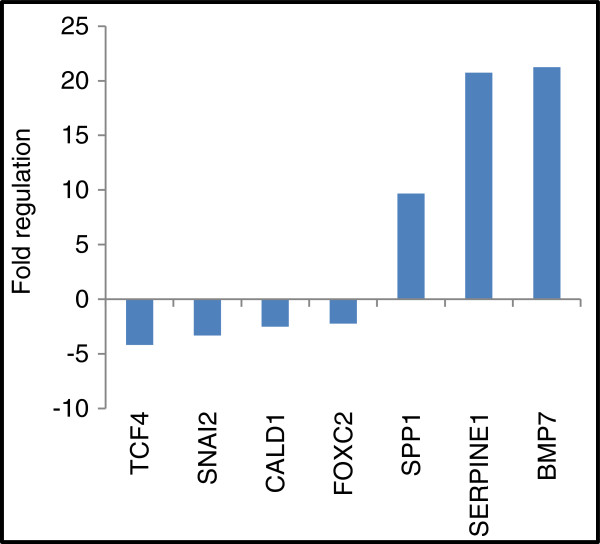
**Silencing of claudin 1 in HBC cells altered expression of genes involved in EMT.** Expression of genes historically linked to EMT was assessed in BT-20 HBC cells in which claudin 1 was knocked down using real-time PCR arrays. Five housekeeping genes were used as controls for each gene expression calculation. Only genes significantly (p<0.02) differentially expressed >2 fold are shown.

PCR array analysis of BT-20 knockdown cells (Table 4) was performed to identify genes whose expressions were altered as a direct consequence of claudin 1 inhibition. Pooled RNA from clone 3 and 4 were used for these analyses. RNA was analyzed in triplicate (three reverse transcription experiments and three qPCR arrays). The results (Table 4) show that the expressions of several genes involved in EMT were significantly altered. Gene expression of SERPINE 1 and SSP1 (osteopontin), two important markers for inhibition of cell migration were significantly up regulated (>20 fold and >9 fold respectively). As well, a significant increase (>20 fold) was observed for BMP7 gene expression, a gene usually associated with cancer progression [30,31]. At the same time, a number of EMT genes; TCF4, SNAIL2 (slug), CALD1 generally associated with maintenance of EMT, were significantly down regulated (Table 4, Figure 6).

**Table 4:** Knockdown of claudin 1 protein in human breast cancer cells resulted in differential expression of EMT related genes

**Symbol**	**Description**	**Fold regulation**
**Up regulated genes**		
BMP7	Bone morphogenetic protein 7	21.23
SERPINE1	Serpin peptidase inhibitor, clade E, member 1	20.75
SPP1	Secreted phosphoprotein 1	9.68
**Down regulated genes**		
TCF4	Transcription factor 4	-4.17
SNAI2	Snail homolog 2 (Drosophila)	-3.31
CALD1	Caldesmon 1	-2.51
FOXC2	Forkhead box C2	-2.23

RNA from 2 control clones were pooled and compared in triplicate with RNA from 2 claudin 1 knockdown clones. Template cDNA samples (25ng) were applied to each real-time PCR reaction on the human epithelial to mesenchymal transition (EMT) PCR array (SA Biosciences). Only those genes whose expression was significantly (p<0.02) differentially expressed (>2 fold) are listed.

## Discussion

Based on the observation that claudin 1 is down regulated or absent in invasive HBC [19-22], and that an absence of claudin 1 was shown to correlate with poor prognosis and shorter patient survival time [23], it has been speculated that claudin 1 could be a putative tumor suppressor in breast cancer. However, these studies, including those from our laboratory, were carried out on breast tumors of mixed pathological lesions. Moreover, when the breast cancers were grouped according to ER status, we observed that not only was the frequency of claudin 1 expression significantly higher in the ER-ve cancers but that a higher level of the protein was also associated with the BLBC subtype; the latter has recently been confirmed by a report by Lu et al., [32] as well as our present study. Additionally, in The Cancer Genome Atlas (TCGA) breast carcinoma provisional dataset, RNAseq analysis has shown claudin 1 to be up regulated in 17/81 (21%) of basal-like tumors compared with 2/324 (<1%) of luminal A/B cases [33]. Since BLBCs are usually mesenchymal in phenotype and high claudin 1 is generally associated with epithelial phenotype, this result was unexpected. However high endogenous claudin 1 levels have also been observed in HBC cell lines as in the case of the BT-20 cell line and several other basal-like cell lines such as HCC1143, and HCC1937 [34]. It is possible that in these breast cancer cells, claudin 1 has a different function.

An important finding of the present study was the significant association between claudin 1 and patient age. BLBC derived from women over 55 years of age were more likely to exhibit high claudin 1 expression. The significance of this observation is not known, but it is plausible that increased claudin 1 levels in these women may be related to decreased hormonal levels generally associated with the post-menopausal stage in a woman's life. As we have previously shown, there is a positive association between claudin 1 expression and ER-ve breast cancers [19]. Thus, the relationship between estrogen and claudin 1 warrants further examination.

The present study also reveals a significant positive relationship between claudin 1 and claudin 4. However, interestingly, no significant association between claudin 4 and patient age was established suggesting that claudin 1 may have a unique role independent of claudin 4.

We also observed that mislocalization of claudin 1 to the cytoplasm was a frequent occurrence in BLBC. Such mislocalization of claudin 1 in the cytoplasm is not unique to breast cancer, as indeed there have been several recent reports of claudin 1 mislocalization in the cytoplasm, and in some cases, the nucleus, in a number of other cancers including melanomas, colon, and oral squamous and colon cancer [11,16-18,35]. In these cancers, claudin 1 mislocalization was shown to increase the invasiveness of the cancer cells [11,16-18,35]. This observation leads us to speculate that it is possible that cytoplasmic claudin 1 may have a different function from membranous claudin 1, as mislocalization of a number of membrane and subcellular proteins to the cytoplasm in some studies has been shown to impart tumorigenicity [36-40].

We showed that stable shRNA knockdown of claudin 1 in BT-20 HBC cells resulted in a subsequent decrease in cell migration and motility. Claudin 1 knockdown also resulted in a significant up regulation of the expression of EMT related genes, SERPINE 1 (plasminogen activator inhibitor type 1, PAI1) and secreted phosphoprotein 1 (SSP1; also known as osteopontin) that have been shown to suppress cancer cell migration. In previous reports, SERPINE 1 was shown to inhibit cell migration during wound healing by blocking integrin from binding to vitronectin [41]. Vitronectin enhances the migration of cells and is required for cell motility [41]. Conversely, SERPINE 1 is also thought to have a role other than a protease inhibitor as it has been shown to decrease the adhesive strength of cells to their substratum. SERPINE 1 is also regulated by a variety of hormones and cytokines [42]. This would be important if in older women, the up regulation of claudin 1 is related to their hormonal status, in particular, the lower estrogen level that is associated with the post-menopausal state. Another gene that was highly up regulated when claudin 1 was suppressed was SSP1. SSP1 is a phosphorylated glycoprotein secreted by several cell types, including those involved in bone turnover and is associated with bone metastasis in cancer [43-45]. It is also secreted by cells of the immune system and is believed to be an early marker for breast cancer [46]. The significant up regulation of these molecules in response to claudin 1 knockdown suggests that claudin 1 may be a regulator of genes associated with cancer progression and metastasis.

At the same time, we observed the down regulation of expression in another group of genes thought to be important for maintaining the EMT phenotype; TCF4, SNAIL2, FOXC2 and CALD1. SNAIL 2, a transcription factor and an important marker of EMT, has been shown to repress both E-cadherin, a master programmer of EMT [47], and claudin 1 [48-52]. TCF4, which belongs to the β-catenin pathway, is a member of the Zeb family of transcription factors. It has been suggested that claudin 1 is a targeted gene of β-catenin. Miwa et al. [53] reported that in squamous cell carcinoma, TCF4 and β-catenin complexes bound TCF4 binding elements at two sites in the 5′ flanking region of the claudin 1 gene and that the binding promoted transcription of claudin 1. As well, SSP1, whose expression is significantly up regulated when claudin 1 is inhibited in this cell line, is a downstream target for TCF4 [54]. TCF4 can act as a promoter or repressor of HBC progression by regulating SSP1 [44,54]. FOXC2 (forkhead box C2), another gene whose expression is significantly down regulated, is a sonic hedgehog (SHH) signaling molecule [55]. Elevated levels of FOXC2 protein have recently been shown to be significantly associated with the BLBC phenotype and with poor disease free survival [55]. Interestingly, SNAIL2, TCF4 and FOXC2 have been identified as part of the E-cadherin repressor interactome in EMT [56] and are involved in many relationships regulating each other in a hierarchical pattern. In this general pathway, it is believed that SNAIL 2 is initially induced, leading to the activation of TCF4 and FOXC2. Also, knocking-down claudin 1 strongly increased the expression of the BMP7 gene, which belongs to one of the largest sub-families of transforming growth factor beta (TGFβ) [57]. TGFβ, itself another important EMT molecule, has a dual role during tumor progression; initially as a suppressor, and then as a promoter. BMP7 is also known to display a number of diverse behaviors with regards to cell proliferation, cell migration, invasion and apoptosis in breast cancer cell lines, primary tumors as well as xenografts [30,31,58-60]. Thus, the influence of claudin 1 on these signaling pathways in the BT-20 HBC cells hints at the complexity of its involvement in cellular processes and tumorigenesis [13,14].

The effect of claudin 1 on cell migration was dose dependent (although not statistically significant). We observed that the rate of migration of clone 3, a clone in which claudin 1 was almost completely knocked down, was slower (p<0.01 when compared with control cells) compared to the other clonal line, clone 4 (p<0.05).

Our earlier studies indicated that tumors which displayed the basal-like phenotype more frequently expressed claudin 1, and were also more likely to express higher levels of claudin 1. Many of these tumors also displayed mislocalization of claudin 1 to the cytoplasm, suggesting that the role of claudin 1 in the breast cancer cell is influenced not only by its level but by its location as well.

Altogether, our studies show that high claudin 1 protein levels are significantly associated with a particular group of older BLBC patients. In this regard, claudin 1 has the potential to serve as a marker for a subset of patients within the BLBC phenotype and in so doing may facilitate more personalized management of this disease. We also show *in vitro* that in basal-like HBC cells, claudin 1 inhibition results in decreased cell migration. Therefore, the expression of high claudin 1 levels in the BLBC subtype, particularly in women over 55 years of age suggests that these patients may warrant more aggressive treatment as their breast cancer may be more migratory resulting in a tendency to move away from the primary location.

## Conclusion

Although there is a growing appreciation for the heterogeneous nature of breast cancer [1], currently, many of the breast cancer subtypes identified remain poorly characterized. A consequence of this lack of biological insight is that the more aggressive subtypes such as the BLBC lead to poorer prognosis, as current therapeutic strategies are mostly ineffective. It is therefore critical to fully delineate the role of structural proteins such as claudin 1 in breast cancer as such knowledge could facilitate more effective patient management. These observations will contribute further to the characterization of this poorly characterized breast cancer subtype, and will enhance our understanding of the paradoxical disease outcome which is often associated with patients with BLBC.

### Consent

Written informed consent was obtained from the patient for publication of this report and any accompanying images.

## Abbreviations

+ve: Positive; -ve: Negative; BLBC: Basal-like breast cancer; CK5/6: Cytokeratin 5,6; EGFR: Epidermal growth factor receptor; EMT: Epithelial-mesenchymal transition; ER: Estrogen receptor; HBC: Human breast cancer; HER2: Human epidermal growth factor receptor 2; IHC: Immunohistochemical; MBTB: Manitoba breast tumor bank; PR: Progesterone receptor; TJ: Tight junction; TMA: Tissue microarray.

## Competing interest

The authors have no known conflicts of interests either financial or personal between themselves and others that might bias the work.

## Authors’ contributions

YM, designed, and supervised the study. YM and AAB co-wrote the manuscript. AAB, and CP carried out H scoring evaluation of the TMAs. XM generated stable clonal cell lines. XM, DM, KD and SC carried out functional assays in the human breast cancer cell lines. KD was also responsible for the subcellular fractionation assays. EL and LCM contributed intellectually to many aspects of the study, and EL reviewed and edited the manuscript. All authors read and approved the final manuscript.

## Pre-publication history

The pre-publication history for this paper can be accessed here:

http://www.biomedcentral.com/1471-2407/13/268/prepub
